# Evidence of a significant vitamin D deficiency among 9–13-year-old Polish children: results of a multicentre study

**DOI:** 10.1007/s00394-018-1756-4

**Published:** 2018-06-23

**Authors:** Danuta Chlebna-Sokół, Jerzy Konstantynowicz, Paweł Abramowicz, Beata Kulik-Rechberger, Marek Niedziela, Anna Obuchowicz, Katarzyna Ziora, Jolanta Karalus-Gach, Joanna Golec, Izabela Michałus, Elżbieta Karczmarewicz, Zenon Piotr Halaba

**Affiliations:** 1 Department of Pediatric Propaedeutics and Metabolic Bone Diseases, Medical University of Lodz, Sporna Street 36/50, 91-738 Łódź, Poland; 2 Department of Pediatric Rheumatology, Immunology, and Metabolic Bone Diseases, Medical University of Bialystok, Waszyngtona Street 17, 15-2742 Białystok, Poland; 3 Departament of Paediatric Propaedeutics, Medical University of Lublin, Gębali Street 6, 20-091 Lublin, Poland; 4 Department of Pediatric Endocrinology and Rheumatology, Poznan University of Medical Sciences, Szpitalna Street 27/33, 60-572 Poznań, Poland; 5 Department of Paediatrics, School of Health Sciences in Katowice, Medical University of Silesia, Batorego Street 15, 41-902 Bytom, Poland; 6 Department of Paediatrics, School of Medicine with the Division of Dentistry in Zabrze, Medical University of Silesia, 3-go Maja Street 13-15, 41-800 Zabrze, Poland; 7 Department of Biochemistry and Experimental Medicine, The Children’s Memorial Health Institute in Warsaw, Aleja Dzieci Polskich 20, 04-730 Warsaw, Poland; 8 Department of Medical Simulation, University of Opole, Oleska Street 48, 45-052 Opole, Poland

**Keywords:** Vitamin D deficiency, 25-Hydroxyvitamin D concentration, Children, Preadolescents, Seasonality

## Abstract

**Purpose:**

To evaluate the extent to which the population of Polish preadolescents is vitamin D deficient and to assess seasonal variations in vitamin D status.

**Participants and methods:**

A total of 720 healthy children aged 9–13 years (409 girls, 311 boys) residing in 6 representative geographical locations in Poland were studied. A parental-assisted questionnaire provided data on nutritional habits, vitamin D supplements and sun exposure. Serum concentration of 25-hydroxyvitamin was determined twice, after the winter in March and after the summer in October.

**Results:**

In March, vitamin D deficiency (25–50 nmol/L) was found in 64%, and severe deficiency (< 25 nmol/L) in 20.2% of children. In October, the deficiency and severe deficiency were still noticed in 25.9 and 0.1% of children, respectively. The mean serum concentration of 25-OHD was 52% higher in October (55.4 ± 14.0 nmol/L) than in March (36.4 ± 13.5 nmol/L), (*p* < 0.01). In children with 25-OHD < 50 nmol/L in March, their 25-OHD concentration increased by 64% through March to October (32.5 ± 8.2 vs. 53.2 ± 7.9 nmol/L, *p* < 0.01). An association was found between 25-OHD concentration and regular consumption of vitamin D supplements, cod-liver oil and fish.

**Conclusions:**

The majority of preadolescent Polish boys and girls show vitamin D deficiency after the winter period, although a distinct amelioration over summertime is found in this age group. There is a need to implement effective prevention and intervention strategies in the management of vitamin D deficiency among schoolchildren in Poland, with the supplementation throughout the entire year.

**Electronic supplementary material:**

The online version of this article (10.1007/s00394-018-1756-4) contains supplementary material, which is available to authorized users.

## Introduction

Over the last few decades, vitamin D status has been extensively evaluated in different populations worldwide, including various ethnic and age groups. A large body of evidence has been published regarding numerous health benefits attributable to vitamin D in adults, the elderly and paediatric populations [1–3]. Vitamin D deficiency in children has been linked to adverse effects such as growth failure and rickets. Adequate levels of vitamin D may also help reduce risk of autoimmune conditions, infection and type 2 diabetes [4]. It is well known that about 90% of vitamin D is produced in the skin by sunlight and only 10% comes from food [5]. However, skin synthesis is not always sufficient to provide the recommended dose in high-latitude regions such as Poland, which is situated between 49° and 54.5° North latitude; therefore, vitamin supplements are usually recommended between October and April, as an alternative for children through the season of limited skin synthesis of vitamin D [6]. Observational studies suggest that populations with a low serum 25-OHD concentration are at risk of chronic conditions, such as cardiovascular diseases, multiple sclerosis, type 1 diabetes, some types of cancer and osteoporosis, although the level of evidence supporting these associations differs between studied populations and published reports [7]. It is well known that vitamin D supply is extremely important for normal development and is crucial not only for the prevention of rickets, but also for appropriate musculoskeletal health during growth, achieving optimal peak bone mass and fracture prevention [8]. Several studies have shown that both adults and children worldwide demonstrate low 25-OHD levels resulting from an inappropriate vitamin D supply, including lifestyle factors, limited exposure to UVB and nutritional deficits [9, 10]. Therefore, a number of current practice guidelines underline the role of nutritional factors and focus on lifestyle characteristics associated with vitamin D deficiency. On the other hand, the differential data from published studies may challenge cutoffs for vitamin D sufficiency and criteria of adequate intakes, leading to a strategy based on a personalized approach, with respect to physiological characteristics, individual requirements and lifestyle [11].

There is a scarcity of data on vitamin D deficiency in Polish children and adolescents and all the existing studies have a cross-sectional design [12]. A recent study, conducted nationally on a large representative population (*n* = 5775), demonstrated evidently that the vast majority of young adults were vitamin D deficient [13]. According to an international survey, the proportion of Polish teenage population below the estimated average requirement for vitamin D was very high (92–100%), with mean intakes ranging from 3.2 µg/day in girls to 4.8 µg/day in boys [14]. In addition, a trend to decline in vitamin D intake was observed among 11–15-year-old Polish children over the period between 1988 and 2006, according to a large study encompassing 9747 randomly selected preadolescent boys and girls [15]. The reported dietary and supplemental intakes of vitamin D ranged between 1.9 and 3.3 µg/day in this survey, whereas official national guidelines of that time recommended at least 10 µg/day from October to March in general healthy population aged 2–18 years [16]. The updated practical guidelines, based on the subsequent Central European consensus, reinforced a more comprehensive strategy, and thus pointed out a need to enhance the recommended daily dose of vitamin D up to 15–25 µg for all prepubertal and adolescent population [17].

The objective of this study was to determine the serum 25-OHD level for preadolescent schoolchildren aged 9–13 years, to assess the prevalence of vitamin D deficiency, and to evaluate the association between some lifestyle and environmental factors (nutrition, supplements, seasonal variations) and 25-OHD concentrations.

## Participants and methods

### Study population

This two-stage study (initial cross-sectional investigation in March and re-examination in October) was conducted nationally in 2011, and included 720 healthy Caucasian children (311 boys, 409 girls) aged 9.0–12.99 years, i.e., individuals attending grades 2–5 of public primary schools with similar physical education curricula. Children residing in six representative geographical locations in Poland were screened for inclusion criteria and enrolled to the survey. The study settings were both urban and rural areas of the following regions: Łódź (51.7°N), Poznań (52.4°N), Lublin (51.2°N), Szczecin (53.4°N), Białystok (53.1°N), the Upper-Silesian Conurbation (50.3°N). Recruitment was based on chronological age criteria, school proximity to research teams, and voluntary parental cooperation. The structured interview was conducted by a nurse with both the child and his/her parents. In all study sites, a pediatrician carried out routine physical examination and recorded medical signs and findings. Based on the initial medical check-up, participants were screened for conditions or medication known to affect bone tissue and/or skeletal health. Children with malabsorption, chronic gastrointestinal diseases, liver diseases, kidney diseases, endocrine disorders and diabetes, eating disorders, apparent metabolic disorders, connective tissue diseases, current or past glucocorticoid use, and therapies with anticoagulants, diuretics or anticonvulsants were excluded from the study.

### Data collection

A parental-assisted questionnaire and a direct interview were used to evaluate dietary intakes of vitamin D supplements, fish-oil and multivitamin preparations, nutritional habits (reports on long-term use of restrictive/elimination diets, i.e., milk-free or gluten-free, vegan/vegetarian diets), level of physical/sport activity (number of hours per week at school, additional sports classes, number of hours of sedentary activity and the child’s leisure time activities). Nutritional intakes were assessed using a modified food frequency questionnaire, based on the last weekday record. Sunlight exposure was estimated according to the method published elsewhere [18]. The calculation method was based on a recall questionnaire evaluating daily time in sun for one typical sunny summer week (< 5, 5–30, > 30 min/day) and was expressed as the number of hours per week spent outdoors. Skin exposure was assessed using four choices: face and hands or face/hands and arms, or face/hands and legs, or bathing suit. A calculation of body surface area (skin area) effectively exposed to sunlight was carried out, and the use of UV sunscreens was analysed (supplementary material). Physical examination and anthropometric measurements were carried out using the same methodology, technical procedures and standardized equipment across the 6 centres. Weight and standing height were measured using conventional methods (electronic scale, Martin stadiometer), and body mass index (BMI; kg/m^2^) was obtained from a standard formula. Table 1 shows the characteristics of the studied group. Blood samples were collected twice in each participating child, to determine serum concentration of 25-hydroxyvitamin D (25-OHD): I—in March (following the winter season), and II—in October (following the summertime). The sera were immediately frozen and stored at − 80 °C and transferred to the central certified laboratory (Department of Biochemistry and Experimental Medicine, Children’s Memorial Health Institute in Warsaw). A double test for 25-OHD concentration was performed with the electro-immuno-chemiluminescent method (ECLIA) using an ELECSYS 2010 analyzer. This method was previously used and validated for routine laboratory procedures, based on the international quality control system (DEQAS); intra-assay CV was less than 8%, whereas the inter-assay CV was less than 12% [19]. The 25-OHD serum concentration was expressed in nanomole per litre (nmol/L).

**Table 1:** Anthropometric characteristics of subjects studied

Variable	Age (years)							
	9.0–9.99 (*n* = 63)		10.0–10.99 (*n* = 259)		11.0–11.99 (*n* = 273)		12.0–12.99 (*n* = 125)	
	Boys	Girls	Boys	Girls	Boys	Girls	Boys	Girls
Weight (kg)	33.2 ± 6.0	34.3 ± 8,4	34.2 ± 5.9	36.8 ± 7.5	40.2 ± 8.4	39.6 ± 9.4	43.0 ± 12.8	44.7 ± 8.5
Height (cm)	137.5 ± 6.8	136.8 ± 8.6	139.6 ± 5.9	141.2 ± 6.7	146.7 ± 7.6	146.6 ± 6.9	150.6 ± 8.7	153.0 ± 6.2
BMI (kg/m^2^)	17.5 ± 2.4	18.1 ± 3.1	17.5 ± 2.5	18.4 ± 2.9	18.6 ± 2.7	18.3 ± 3.3	18.7 ± 4.4	19.0 ± 3.1
25-OHD (nmol/L)								
In March	36.7 ± 13.2	42.2 ± 15.5	40.9 ± 14.0	41.4 ± 18.5	37.4 ± 14.5	35.4 ± 12.5	34.4 ± 12.7	36.7 ± 13.7
In October	59.2 ± 9.2	55.2 ± 11.7	58.4 ± 17.2	50.0 ± 10.7	54.9 ± 12.5	47.4 ± 11.2	55.7 ± 14.5	45.7 ± 7.5
*p* value March vs. October	< 0.01	0.08	< 0.000	< 0.000	< 0.000	< 0.000	< 0.004	< 0.002

Mean values ± SD are shown

### Data analysis

Adequate levels of 25-OHD were considered as those ranging from 50 to 200 nmol/L, deficiency was assigned to a level less than 50 nmol/L, based on the Practical Guidelines for the supplementation of vitamin D and the treatment of deficiency in Central Europe [17]. Prespecified thresholds were used to show varying degrees of 25-OHD deficiency (< 50, < 25 nmol/L), and severe deficiency was set at the level lower than 25 nmol/L.

All calculations were done using Statistica 10 (StatSoft, Inc., Tulsa, USA). The data were expressed as mean and standard deviation. For comparison between the levels of 25-OHD in relation to seasonality and gender, the Mann–Whitney test was applied. In the case of dependent nominal variables for statistic significance assessment, the McNemar test and, for independent variables, the Pearson *χ*^2^ test was used. For small size samples, Yates’ correction or Fisher’s exact test were utilised. Significance was determined at *p* level < 0.05.

## Results

### Vitamin D status

**Fig. 1:**
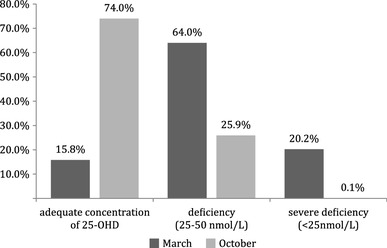
Proportion of children with adequate and inadequate vitamin D levels in relation to the season of the year

The serum concentration of 25-OHD below 50 nmol/L after winter (end of March) was found in 606 of all 720 children (84.2%). Among them, 461 (64%) had a concentration ranging between 25 and 50 nmol/L, whereas 145 individuals (20.2%) had less than 25 nmol/L. Adequate levels of 25-OHD were observed in only 114 children (15.8%) (Fig. 1). The region-specific analysis of 25-OHD status in March showed that the lowest proportions of vitamin D deficient and severely deficient children were found in central Poland (Łódź and Poznań), where 77 and 74% of the population, respectively, demonstrated 25-OHD concentrations below 50 nmol/L. In other regions, the deficiency was found in similar proportions and ranged from 88 to 95% of the studied children. After the summer (measurement in October), 25-OHD levels showed a significant improvement across the country. An increase of 25-OHD was observed regardless of the geographical region, particularly in those children who had previously demonstrated severely deficient levels (< 25 nmol/L). However, deficiency was still found in 26% of all studied children (Fig. 1).

### Predictors for vitamin D status and season-specific effect

No significant differences in serum 25-OHD concentration were observed between boys and girls, nor was this finding influenced by age. However, a significant season-specific effect on vitamin D status was found in nearly all studied children, i.e., a distinct increase in 25-OHD was observed from March to October, showing a relationship between summer and vitamin D supply (Table 1). The mean serum concentration of 25-OHD was 52% higher in October (55.4 ± 14.0 nmol/L) than in March (36.4 ± 13.5 nmol/L), (*p* < 0.01). In children with 25-OHD < 50 nmol/L in March, their 25-OHD concentration increased by 64% through March to October (32.5 ± 8.2 vs. 53.2 ± 7.9 nmol/L, *p* < 0.01). A scant proportion of participants reported appropriate seasonal supplementation with vitamin D in winter. Regular use of supplements containing vitamin D (400 IU/day) for an average of 3–4 months was reported by 42 (6%), multi-vitamins (100–200 IU of vitamin D per day) by 171 (23%), and fish-oil (200 IU of vitamin D per day) by 197 (27%) of the studied children. On the other hand, data also showed that a larger proportion of the children with adequate 25-OHD levels received vitamins in comparison with children who had deficient 25-OHD levels (Table 2). Analyses of the leisure time spent outdoors and the area of skin exposed to sunlight/UVB during summer revealed that children with adequate levels of 25-OHD in October reported a greater sun exposure and a higher number of hours spent outdoors in summer in comparison with vitamin D deficient and severely deficient children (Table 3). Furthermore, it was demonstrated that a larger proportion of children with sufficient serum 25-OHD in October spent their holidays in Mediterranean countries, compared to those with deficient levels (74 and 26%, respectively).

**Table 2:** Comparison of self-reported intakes of vitamin D from specific dietary sources in children with deficient and adequate levels of serum 25-OHD measured after winter

The source of vitamin D supplementation	Participants with the level of 25-OHD < 50 nmol/L		Participants with the level of 25-OHD ≥ 50 nmol/L		*p* value
	Supplemented, *n* (%)	Not supplemented, *n* (%)	Supplemented, *n* (%)	Not supplemented, *n* (%)	
Cod-liver oil	147 (27%)	399 (73%)	46 (46%)	54 (54%)	< 0.01
Multi-vitamins	129 (23%)	422 (77%)	39 (39%)	62 (61%)	< 0.01
Vitamin D only products	31 (6%)	495 (94%)	11 (12%)	81 (88%)	< 0.03

*p* values are derived from *χ*^2^ test

**Table 3:** Comparison of outdoor activities and skin exposure to UVB in children with deficient and adequate levels of 25-OHD measured after summer

Time spent outdoor (h/week)	Children with the level of 25-OHD < 50 nmol/L, *n* (%)	Children with the level of 25-OHD ≥ 50 nmol/L, *n* (%)	The body surface area exposed to UVB (%)	Children with the level of 25-OHD < 50 nmol/L, *n* (%)	Children with the level of 25-OHD ≥ 50 nmol/L, *n* (%)
Less than 2	7 (52.9%)	6 (46.1%)	< 10	5 (50%)	5 (50%)
2–6	88 (40.7%)	128 (59.3%)	10–50	122 (37.9%)	200 (62.1%)
7–10	64 (26.8%)	175 (73.2%)	> 50	29 (23.4%)	95 (76.6%)

A significant improvement in vitamin D status found in the overall population at follow-up (re-examination in October) proved the beneficial effect of the time spent outdoors, i.e., exposure to sunlight. Skin synthesis of vitamin D was presumably most effective in the children with severe deficiency, as the majority of them demonstrated a proportional increase in concentration, reflecting a complete amelioration.

## Discussion

Our study included a representative sample of prepubertal children from different regions of Poland and clearly showed a deficiency of 25-OHD in this age group. We also extended our observation by demonstrating distinct seasonal variations in vitamin D status. The prevalence of vitamin D deficiency after winter reached 84.2% in all regions of the country, with the highest rates found in children living in Szczecin and Białystok (Northern Poland), as well as in the Silesian and Lublin regions. The prevalence of vitamin D deficiency after summer was 26% countrywide, with the highest rates in the regions of Łódź, Poznań and Szczecin.

Skin synthesis of vitamin D, mediated through exposure to UVB, was most evident in the children with severe deficiency, as in the majority of these children a significant improvement of the vitamin D status was observed. The considerable proportion of children with severe vitamin D deficiency (20.2% of all participants) was similar to those reported in other countries [20, 21]. Kim et al. in a recent study among healthy Korean adolescents (data from the Korean National Health and Nutrition Examination Survey), demonstrated that 54.7% of teenagers had vitamin D deficiency, and as many as 13.4% had a severe deficiency [20]. The children studied by these investigators also had the lowest 25-OHD levels after winter and in early spring, thereby showing a similar pattern of seasonal variations. Another study of Asian adolescents also reported the largest deficiency rates, up to 89% of the population, observed in spring, with a distinct alleviation up to 63.9% after summer [21]. Consequently, these data indicate poor management and ineffective supplementation in the period when skin synthesis is absent due to geographical factors and latitude. Some other reports comprehensively explain the relationship between vitamin D status, latitude and sunlight exposure. The Brazilian investigators Santos et al. [22] did not observe variability in 25-OHD levels in summer and winter, presumably because the geographic latitude of the area in which they conducted their studies was below 35 degrees, so that dermal synthesis of vitamin D was possible throughout the whole year. Nevertheless, in as many as 54% of girls, vitamin D deficiency (25-OHD < 50 nmol/L) was observed because of the lack of supplementation, low vitamin D diet and overuse of sunscreens. Moreover, Pekkinen et al. found that 71% of Finnish children aged 7–11 years were vitamin D deficient (25-OHD < 50 nmol/L), even despite having met or exceeded the recommended daily intakes for vitamin D [23].

Our results may suggest suboptimal supply of vitamin D among Polish children, though the causality is difficult to demonstrate. Noticeably, according to the Polish official guidelines established in 2009, vitamin D deficiency is considered at the serum 25-OHD concentration below 50 nmol/L [24]. Although this criterion is consistent with the great majority of international recommendations on vitamin D, and several reports have considered a threshold value for adequate 25-OHD levels being 50 nmol/L, there are still controversies and country-specific differences regarding the definition of deficiency or appropriate requirements [11]. Thus, assuming that the level of 75 nmol/L is often believed to be desirable, a greater proportion would be classified as deficient at a higher cutoff.

In the present study, no significant differences in the 25-OHD levels between girls and boys were revealed, consistently with the recent observations by Karonova et al. [25], but contradictory to reports from South Korea [20], the United Kingdom [26] and Finland [23], which found lower 25-OHD levels in girls than in boys. In Canadian children aged from 2 to 16 years, a higher prevalence of vitamin D deficiency in boys compared to girls was also reported [27]. According to this report, 69% of boys and 35% of girls aged 9–16 were vitamin D deficient, while in children aged 2–8 it reached 22 and 8%, respectively. Many investigators emphasize that the prevalence of vitamin D deficiency in childhood and adolescence increases with age [20, 28]. For example, Roth et al. [27] showed that 25-OHD concentrations decrease with age only in boys, whereas Willis et al. [29] found such an association also in girls. In our study, serum concentration of 25-OHD was not influenced by participants’ chronological age. This discrepancy may partly result from increased requirements for vitamin D due to the pubertal growth spurt and the rapid accrual of skeletal mass. On the other hand, teenage children, mainly girls, less frequently comply with dietary recommendations regarding nutritional vitamin D intake [30]. Furthermore, there may be a negative effect of reduced leisure time spent outdoors on the levels of 25-OHD during puberty and adolescence, because as children get older, their outdoor activity and solar UV dose are reduced. This has been reported by other investigators and seems to be typical for most European and also US populations during growth [31].

In our study, we attempted to assess the effects of sun exposure (hours per week and skin area exposed during summer) on serum concentration of 25-OHD. The re-examination in October indicated that children with adequate levels of 25-OHD reported higher UVB exposure and spent more time outdoors in summer, in comparison to children with vitamin D deficiency. This remains consistent with other observations, including McGillivray et al. [32] and Tangpricha et al. [33], who point out the low prevalence of vitamin D deficiency after summer, and highlight the beneficial effects of sun exposure on bone mineralisation. Similar results supporting our findings have been shown in studies evaluating the degree of sun exposure, not only using a questionnaire-based design, but also with the use of dosiometry [34]. Another study conducted in Spanish girls analysed the correlation between the rate of sun exposure in autumn and the wintertime vitamin D levels, demonstrating the beneficial effect of sunlight, in ensuring optimal vitamin D status (mean 25-OHD concentration was 100 nmol/L) [35]. The results of these studies still confirm the vital significance of sun exposure in achieving an adequate supply of vitamin D, and also indicate that supplementation with this vitamin is necessary in cases where effective skin biosynthesis is not possible. In our study, supplementation with vitamin D between October and March was reported by a small number of individuals, despite clear official national guidelines recommending a supply at the amount of 400 IU per day to all children aged 2–18 years [24]. In the literature, there are few studies assessing adherence with supplementation guidelines for the population during growth. Haran et al. conducted a survey among healthcare professionals regarding vitamin D supply to their own children; the supplementation level was found to be very low relative to the routine administration guidelines [36]. Similar observations indicating low rates of vitamin D supplement use were shown in British and Finnish reports [37, 38]. It should be emphasised that in our study, fish-oil and multivitamin preparations were the most commonly administered vitamin D-containing sources, whereas only a small proportion of children had received products containing only vitamin D. Although vitamin D-only products are presumably less frequently recommended by family physicians and paediatricians during growth, consumption of fish-oil and composite vitamins preparations may still have met the requirements. A similar view is also reflected in other studies [37]. Furthermore, there may be a suspicion that, despite receiving many recommendations from their physicians, parents appear non-compliant regarding this aspect of paediatric care. The benefit of fish-oil (cod-liver oil) may result from the fact that this nutritional supplement is widely promoted and accepted as a “healthy” product for children in Poland and, perhaps for this reason, the use of cod-liver oil in the autumn-winter-early spring season was more regular among our study participants. Furthermore, it is important to state that a significantly higher proportion of children whose parents reported supplementation with vitamin D in winter had an appropriate vitamin D status. This finding confirms the rationale for, and the efficacy of, prevention of the deficiency using regular supplementation. This remains in accordance with other observations, and proves that further promotion of vitamin D is needed in the critical period of prepuberty and puberty [39].

There were some limitations of our study that need to be mentioned. The socio-economic status has not been provided in this study. However, we did examine parental education, which was found to have no effect on vitamin D status in their children. Additionally, there are no essential differences, particularly regarding lifestyle, between rural and urban schoolchildren across the country nowadays. We did not analyse potential differences between the study locations. We are aware that the geographical context may be very important indeed, although Poland is not considered to be a large geographical area in terms of potential differences in latitude, exposure to the sun or, for instance, fish consumption.

In summary, prepubertal children in Poland demonstrate significant deficiency of vitamin D, particularly during winter, which remains consistent with most paediatric studies published elsewhere. We conclude that there is a positive effect of seasonal exposure to UVB on 25-OHD blood concentration, suggesting the efficacy of sunlight in correcting the deficits. A significant increase in vitamin D status after summer is likely to result from intensive skin synthesis associated with outdoor activity and habitual sun exposure. Thus, health professionals should actively encourage schoolchildren to modify their leisure time in summer. On the other hand, considering environmental factors (weather alterations, seasonal variations), supplementation with vitamin D is necessary in Polish children throughout the entire year, particularly when skin biosynthesis is limited.

## Electronic supplementary material

Below is the link to the electronic supplementary material.

Supplementary material 1 (DOCX 37 KB)
