# Supplementary material for: Evidence of a significant vitamin D deficiency among 9–13-year-old Polish children: results of a multicentre study
Source: Eur J Nutr. 2018 Jun 23;58(5):2029–36. doi: 10.1007/s00394-018-1756-4 (PMC6647701; doi:10.1007/s00394-018-1756-4)
Supplement: Supplementary file 1 — Supplementary material 1 (DOCX 37 KB) [file 394_2018_1756_MOESM1_ESM.docx]

|  | Time Outdoors | | | Amount of Skin Exposed | | | |
| --- | --- | --- | --- | --- | --- | --- | --- |
|  | < 5 min | 5-30 min | > 30 min | Hands and Face (<10%) | Hands, face, arms (10-50%) | Hands, face, legs (>50%) | Bathing suit (>50%) |
| Monday  Tuesday  Wednesday  Thursday  Friday  Saturday  Sunday |  |  |  |  |  |  |  |

The weekly sun exposure recall questionnaire
